# CircYIPF6 regulates glioma cell proliferation, apoptosis, and glycolysis through targeting miR-760 to modulate PTBP1 expression

**DOI:** 10.1515/tnsci-2022-0271

**Published:** 2023-08-11

**Authors:** Dan Lei, Wenyong Xiao, Bo Zhang

**Affiliations:** Department of Neurosurgery, Hanyang Hospital Affiliated to Wuhan University of Science and Technology, Wuhan, Hubei, 430050, China; Department of Oncology, The Central Hospital of Huangshi, No. 141, Tianjin Road, Huangshigang District, Huangshi City, Hubei, China

**Keywords:** glioma, circYIPF6, miR-760, PTBP1, proliferation, apoptosis, glycolysis

## Abstract

**Background:**

Recent studies have highlighted that circular RNAs regulate cancer-related genes’ expression by functioning as microRNA sponges in cancers. Herein, we investigated the function and molecular mechanism of circYIPF6 in glioma.

**Methods:**

5-Ethynyl-2′-deoxyuridine assay, colony formation, and flow cytometry were performed to assess the proliferation and apoptosis of glioma cells. The levels of glycolytic metabolism were evaluated by measuring the glucose uptake and lactate production. The protein levels of Bax, Bcl2, GLUT1, LDHA, and PTBP1 were examined by western blot. The interplay between miR-760 and circYIPF6 or PTBP1 was confirmed by a dual-luciferase reporter. The effect of circYIPF6 silencing on the growth of glioma *in vivo* was determined by a xenograft experiment.

**Results:**

circYIPF6 was significantly upregulated in glioma. Knockdown of circYIPF6 suppressed glioma cell proliferation and glycolysis while promoting cell apoptosis. Mechanistic studies revealed that circYIPF6 targeted miR-760 and could abundantly sponge miR-760 to inhibit the expression of its downstream target gene PTBP1. Functional rescue experiments showed that both miR-760 inhibition and PTBP1 overexpression could attenuate the regulatory effect of circYIPF6 silencing on glioma cells. Furthermore, circYIPF6 knocking down effectively impeded glioma growth *in vivo*.

**Conclusion:**

These findings suggested that circYIPF6 participated in the proliferation, apoptosis, and glycolysis of glioma through the miR-760/PTBP1 axis.

## 1 Introduction

Glioma is one type of intracranial tumors deriving from the glial cells with a high fatality ratio and poor prognosis [1,2]. Despite significant advances in diagnosis and treatment, the median survival rate of glioma patients is dismal [3]. Circular RNAs (circRNAs) have been found to play a vital role in tumorigenesis and the development of glioma, which can be employed as a novel direction for glioma-targeted therapy [4,5].

circRNAs are highly stable and conserved in mammalian tissues and cells, which have been proven to be a non-coding RNA (ncRNA) with regulatory functions [6]. Thus, circRNAs have become a research hotspot in the area of ncRNA. An increasing number of studies reported that circRNAs were abnormally expressed in cancers and could play a crucial part in cell proliferation, apoptosis, invasion, and energy metabolism through serving as microRNA (miRNA) sponges [7,8,9]. Lyu et al. reported, that circYIPF6 (hsa_circ_0004379) may serve as an oncogene in glioma [10]. However, its role in glioma has not been clarified.

Polypyrimidine tract-binding protein 1 (PTBP1) is an important protein in the regulation of alternative splicing and mRNA metabolism [11]. Previous studies have shown that PTBP1 overexpression accelerated the growth and cell cycle of breast cancer and colon cancer [12,13]. Furthermore, PTBP1 was thought to be an oncogene in multiple myeloma and regulated the expression of aerobic glycolysis genes [14]. In glioma, PTBP1 was found upregulated and involved in the tumorigenesis of glioma [15]. These results suggest that PTBP1 may be an effective target molecule for tumor therapy.

Here, we clarified circYIPF6 expression in glioma and revealed the molecular mechanism by which circYIPF6 regulated PTBP1 level via sponge miRNA and thus regulated cell proliferation, apoptosis, and glycolysis of glioma cells.

## 2 Materials and methods

### 2.1 Tissue sample collection

Tissue samples, including 57 glioma tissues and 43 normal brain tissues, were collected from Hanyang Hospital Affiliated to Wuhan University of Science and Technology.

### 2.2 Cell culture

Human normal astrocytes cell (HA) and glioma cells lines (U251, LN229, TJ905, and SHG44) were got from the China Center for Type Culture Collection (Wuhan, China) and cultured in 10% fetal bovine serum dulbecco’s modified eagle medium (Gbico-BRL, Grand Island, NY, USA).

### 2.3 Quantitative real-time polymerase chain reaction (qRT-PCR)

Extraction of RNA was carried out with TRIzol methods (QIAGEN, Hilden, Germany). Then, the PrimeScript™ RT reagent Kit (TaKaRa, Shiga, Japan) was employed to reversely transcribe the RNA samples. Next, qPCR was conducted using the SYBR Green qRT-PCR kit (QIAGEN). circYIPF6, miR-760, and PTBP1 mRNA expressions were analyzed by 2^−ΔΔct^ method and normalized to GAPDH or U6. The primers are presented in Table 1.

**Table 1:** Primers sequences used for qRT-PCR

Name		Primers for qRT-PCR (5′–3′)
circYIPF6	Forward	GGCTGGTACTTTTGGCTGAT
	Reverse	GGGGATGTCTTGTGAGATGG
YIPF6	Forward	GAGAAGGAGGGCCAAGATGG
	Reverse	AGCTGTCAAACTCCCGGATG
miR-760	Forward	GTATGACGGCTCTGGGTCTG
	Reverse	CTCAACTGGTGTCGTGGA
PTBP1	Forward	TTTTCCAAGCTCACCAGCCT
	Reverse	TATACCAGGTGCACCGAAGG
U6	Forward	CTCGCTTCGGCAGCACATA
	Reverse	CGAATTTGCGTGTCATCCT
GAPDH	Forward	AAGGCTGTGGGCAAGGTCATC
	Reverse	GCGTCAAAGGTGGAGGAGTGG

### 2.4 Cell transfection

The shRNA targeted circYIPF6 (sh-circYIPF6) and non-sense sh-NC were constructed by RIBOBIO (Guangzhou, China). miR-760 mimics (miR-760), miR-760 inhibitors (anti-miR-760), and their control mock (miR-NC, anti-miR-NC), together with pcDNA3.1-PTBP1 and pcDNA3.1 control vector (pcDNA-NC) were offered by Sangon Biotech (Shanghai, China) and then transfected glioma cells for 48 h.

### 2.5 5-Ethynyl-2′-deoxyuridine (EdU) staining and colony formation assay

1 × 10^5^ cells were incubated with EdU (Beyotime) for 2 h in 96-well plates, then 4% paraformaldehyde (Beyotime, Shanghai, China) was used in each well to fix for 30 min after that cell samples suffered the staining using an EdU kit (Beyotime). Images were taken and the EdU-positive rate was calculated.

For the colony formation assay, we plated cells (500/well) into six-well culture dishes and cultured for 10–15 days. Then, 4% polyformaldehyde was applied to fix cells and GIMSA (Merck, Darmstadt, Germany) was employed for staining. Colonies were counted manually.

### 2.6 Flow cytometry for cell cycle and apoptosis

A cell cycle kit (Beyotime) and Annexin V-FITC/PI kit (Solarbio, Beijing, China) were utilized to analyze the cell cycle and apoptosis, respectively. After processing, the cells were analyzed using a FACSCalibur flow cytometer (BD Biosciences, San Jose, CA, USA).

### 2.7 Glucose uptake and lactate production assay

Glucose uptake and lactate production levels were assessed by a glucose uptake colorimetric assay kit and a lactate assay kit (BioVision, Milpitas, CA, USA).

### 2.8 Western blot assay

Protein samples were prepared using RIPA lysate (Thermo Fisher Scientific, Waltham, MA, USA), resolved via sodium dodecyl sulfonate-polyacrylamide gel electrophoresis, and then transferred to PVDF membrane (Millipore, Billerica, MA, USA). Next, membranes were incubated with primary antibody (including antibody against Bax [ab32503; 1:1,000; Abcam, Cambridge, UK], Bcl2 [ab32124; 1:1,000; Abcam], glucose transporter protein 1 [GLUT1, ab115730; 1:1,000; Abcam], lactate dehydrogenase A [LDHA, ab52488; 1:1,000; Abcam], and PTBP1 [ab133734; 1:1,000; Abcam]) followed by incubation with HRP-tagged secondary antibody (Abcam). Anti-β-actin antibody was used as a control. The blots were detected by an ECL kit (Beyotime)

### 2.9 Dual-luciferase reporter gene assay

Wild-type fragments of circYIPF6 and PTBP1 3′UTR containing miR-760 binding sites (circYIPF6-WT and PTBP1 3′UTR-WT) and corresponding mutant sequences (circYIPF6-MUT and PTBP1 3′UTR-MUT) were amplified and then constructed luciferase reporter gene vector. Next, the vectors were co-transfected with miR-760 or miR-NC into cells, respectively. At last, we detected the luciferase activity in cells.

### 2.10 RIP and RNA pull-down assay

The binding of circYIPF6 and miR-760 was determined using the Magna RIP kit (Millipore). The cell lysates were hatched with magnetic beads coupled with an Ago2 antibody or a control IgG antibody. The circYIPF6 and miR-760 enrichment levels in immunoprecipitate were examined by qRT-PCR.

For RNA pull-down assay, biotin-tagged miR-760 was synthesized by RIBOBIO and transfected into U251 and LN229 cells. Then, M-280 streptavidin magnetic beads (Invitrogen) were applied to pull down the biotin-miR-760-circYIPF6 complexes. The bound RNAs were purified by TRIzol, and the circYIPF6 level was measured by qRT-PCR.

### 2.11 Xenograft experiment

Ten Balb/c nude mice were supplied by Shanghai Laboratory Animal Center (Shanghai, China) and employed for the animal experiment, with the approval from the Animal Ethics Committee of Hanyang Hospital Affiliated to Wuhan University of Science and Technology. Mice were injected with U251 cells (1 × 10^7^) transfected with sh-circYIPF6 or sh-NC (*n* = 5), respectively. The tumor volume was observed every 5 days. The mice were euthanatized after injection for 30 days, and the tumors were taken out and weighed. qRT-PCR was applied to measure circYIPF6 and miR-760 expression in xenograft tumors, and the levels of PTBP1, GLUT1, and Ki67 were analyzed by IHC [16].

### 2.12 Statistical analysis

All experiments above were executed in triplicate. The data were analyzed and plotted with the GraphPad Prism 8.0 software. All measurement data were conformed to the normal distribution and presented as mean ± standard. *t* test or one-way analysis of variance was used for comparing differences between two or multiple groups. *P* < 0.05 indicates statistically significant.


**Informed consent:** Informed consent has been obtained from all individuals included in this study.
**Ethical approval:** The research related to human use has been complied with all the relevant national regulations, institutional policies, and in accordance the tenets of the Helsinki Declaration and has been approved by the authors’ institutional review board or equivalent committee. The research obtained the approval from Ethics Committee of Hanyang Hospital Affiliated to Wuhan University of Science and Technology.

## 3 Results

### 3.1 CircYIPF6 is upregulated in glioma tissues and cells

**Figure 1:**
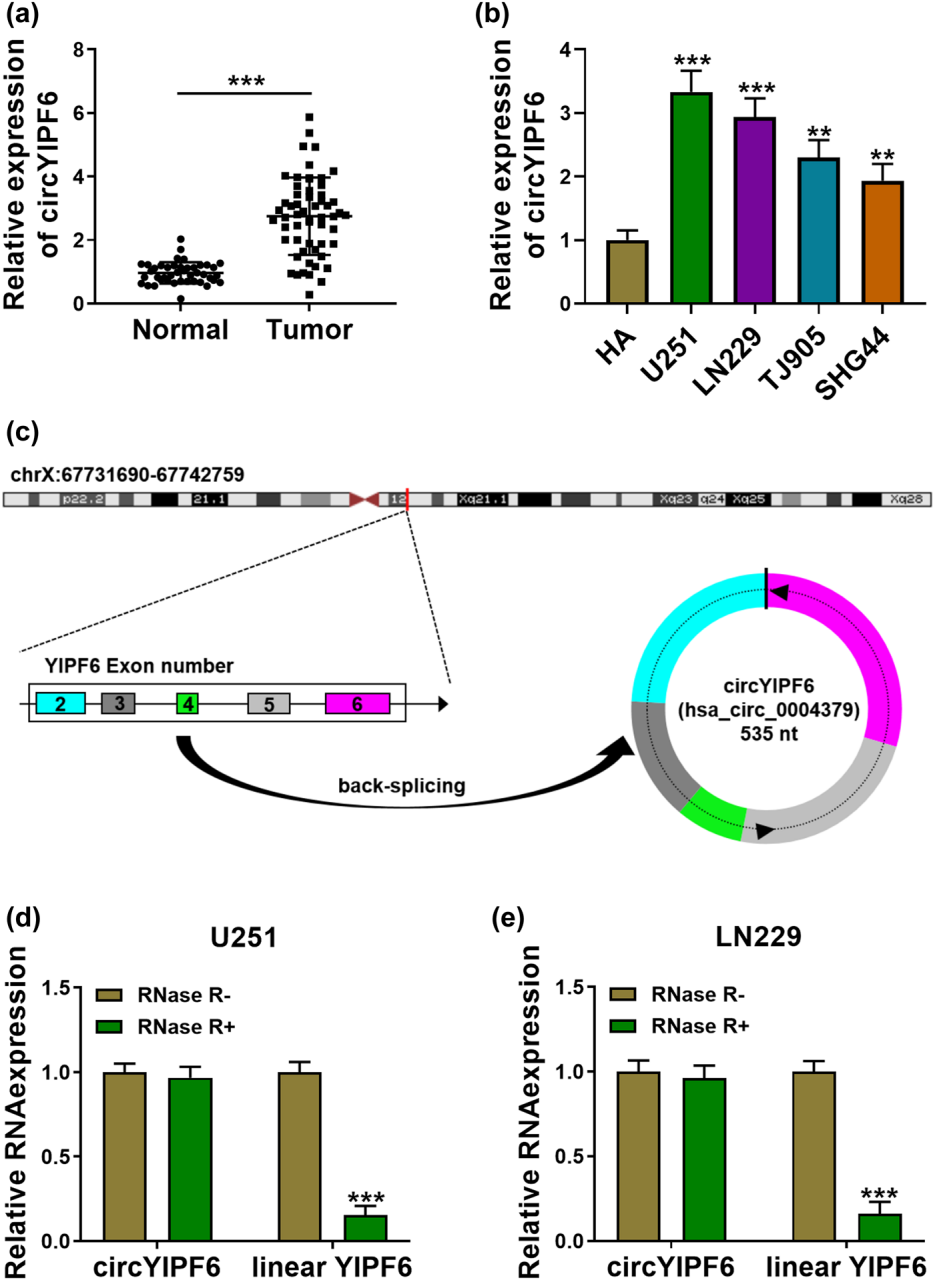
circYIPF6 is highly expressed in glioma tissues and cells. (a) The expression of circYIPF6 in glioma tissues (*n* = 57) and normal brain tissues (*n* = 43) was detected by qRT-PCR. (b) The expression of circYIPF6 in glioma cells (U251, LN229, TJ905, and SHG44) and human normal astrocytes (HA) was detected by qRT-PCR (*n* = 3). (c) Genomic location and formation of circYIPF6. (d and e) qRT-PCR for the expression of circYIPF6 and linear YIPF6 in glioma cells treated with RNase R (*n* = 3). ***P < 0.001, **P < 0.01.

To clarify the expression patterns of circYIPF6 in glioma, we performed qRT-PCR and found that circYIPF6 was highly expressed in 57 glioma tumor tissues compared with 43 normal brain tissues (*P* < 0.001, Figure 1a). Consistently, circYIPF6 was also upregulated in glioma cell lines (U251, LN229, TJ905, and SHG44) (*P* < 0.01, Figure 1b). Bioinformatics software of circRNAs (CircView) showed that circYIPF6, located in chrX:67731690-67742759, was an exonic circRNA that was cyclized with the 2, 3, 4, 5, and 6 exons of the YIPF6 gene and the spliced length of circYIPF6 was 535 nt (Figure 1c). RNase R and qRT-PCR were applied to distinguish circYIPF6 from linear YIPF6. The results showed that linear YIPF6 was digested by RNase R, but not circYIPF6 (*P* < 0.001, Figure 1d and e). These results showed that circYIPF6 was highly and stably expressed in glioma.

### 3.2 Knockdown of circYIPF6 inhibits glioma cell proliferation and glycolysis and promotes cell apoptosis *in vitro*

**Figure 2:**
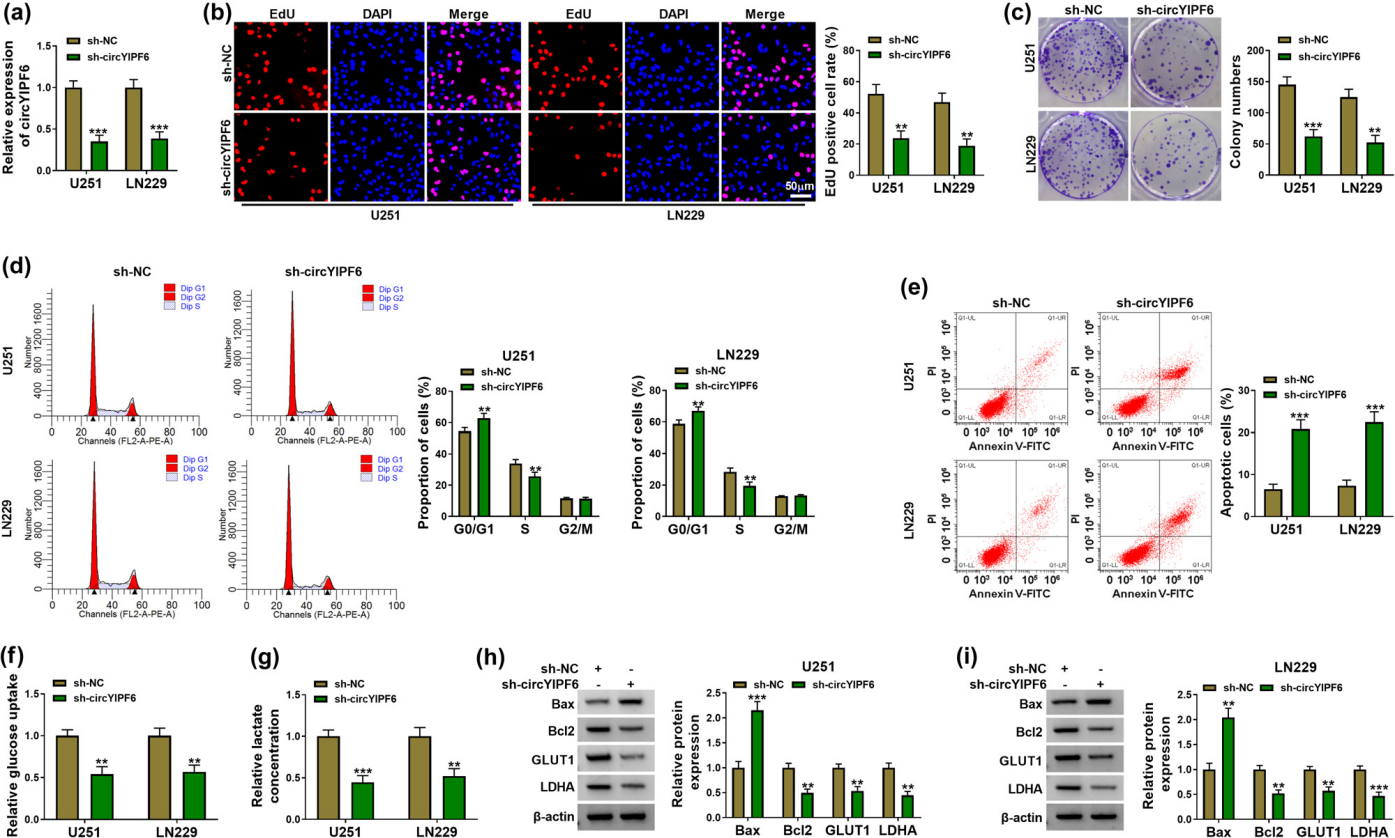
Silencing of circYIPF6 suppressed the proliferation and glycolysis and promotes apoptosis of glioma cells *in vitro*. (a) qRT-PCR detection of circYIPF6 in glioma cells transfected with sh-circYIPF6 or sh-NC (*n* = 3). (b) Representative images (left) and EdU-positive cell rate (right) in glioma cells transfected with sh-circYIPF6 or sh-NC of the EdU staining assay (*n* = 3). (c) Colony formation images (left) and colony numbers statistics (right) in glioma cells transfected with sh-circYIPF6 or sh-NC (*n* = 3). (d) Flow cytometry detection for cell cycle distribution in glioma cells transfected with sh-circYIPF6 or sh-NC (*n* = 3). (e) Flow cytometry detection for cell apoptosis in glioma cells transfected with sh-circYIPF6 or sh-NC (*n* = 3). (f and g) The glucose uptake and lactate concentration in glioma cells transfected with sh-circYIPF6 or sh-NC were examined (*n* = 3). (h and i) Western blot indicating the expression of Bax, Bcl2, GLUT1, and LDHA in glioma cells transfected with sh-circYIPF6 or sh-NC (*n* = 3). ***P < 0.001, **P < 0.01.

To evaluate the biological functions of circYIPF6 in glioma, we first established circYIPF6-knockdown glioma cell lines by using shRNA targeting circYIPF6 to transfect U251 and LN229 cells, respectively. circYIPF6 was decreased in cells transfected with sh-circYIPF6 (*P* < 0.001, Figure 2a). EdU staining and colony formation assay revealed that knocking down circYIPF6 signally suppressed the proliferative activity of glioma cells (*P* < 0.01, Figure 2b and c). The results of flow cytometry showed that circYIPF6 knockdown induced G0/G1 arrest in cells (*P* < 0.01, Figure 2d) and promoted the apoptosis ratio (*P* < 0.001, Figure 2e). Furthermore, the glucose uptake and lactate production were reduced in cells with circYIPF6 knockdown (*P* < 0.01, Figure 2f and g). The detection of apoptosis-related proteins (Bax and Bcl2) and glycolytic-related proteins (GLUT1 and LDHA) showed that circYIPF6 knockdown increased the abundance of Bax and decreased the level of Bcl2, GLUT1, and LDHA (*P* < 0.01, Figure 2h and i). These results suggested that circYIPF6 knockdown played an inhibitory effect on glioma cell progression.

### 3.3 CircYIPF6 directly targets miR-760 in glioma cells

**Figure 3:**
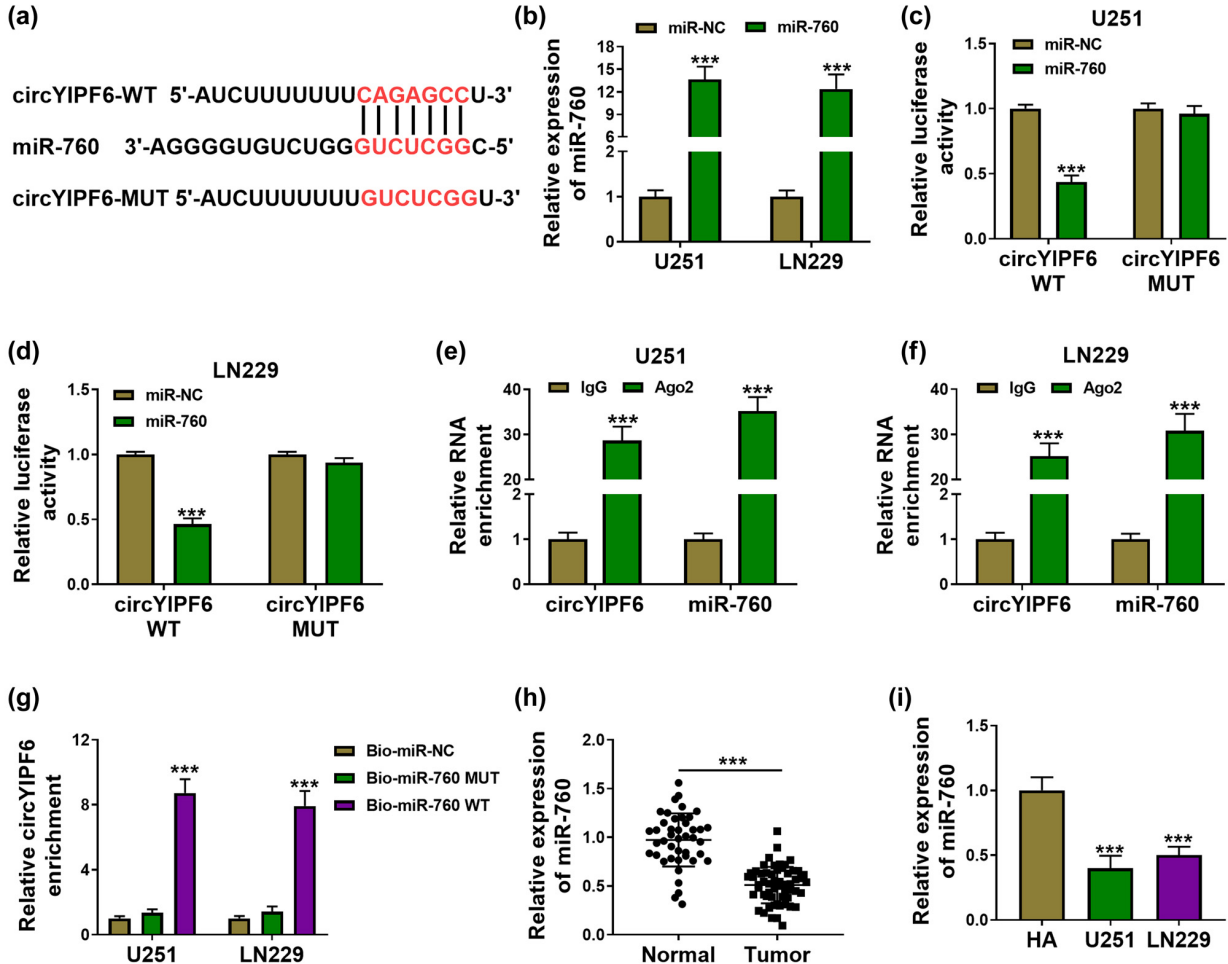
circYIPF6 serves as a sponge for miR-760. (a) The predicted miR-760 binding sites in the circYIPF6. (b) qRT-PCR detection of miR-760 in glioma cells transfected with miR-760 mimics or miRNA mimics (*n* = 3). (c and d) Luciferase reporter activity of circYIPF6-WT or circYIPF6-MUT in glioma cells after transfection with miR-760 (*n* = 3). (e and f) Fold enrichment of circYIPF6 and miR-760 by Ago2 antibody or IgG antibody in glioma cells (*n* = 3). (g) The enrichment of circYIPF6 by miR-760 pull-down in glioma cells (*n* = 3). (h and i) The expression of miR-760 in tumor tissues (*n* = 57) and normal tissues (*n* = 43) and cells (*n* = 3) were detected by qRT-PCR. ***P < 0.001.

Bioinformatics database starBase was applied to predict miRNAs, which could bind with circYIPF6. Among them, we were interested in miR-760, which has been reported to be underexpressed in glioma [17]. The predicted binding sites between circYIPF6 and miR-760 are displayed in Figure 3a. Transfection of miR-760 mimics successfully generated an increased miR-760 expression in cells (*P* < 0.001, Figure 3b). The luciferase activity of the circYIPF6 WT vector (*P* < 0.001) was significantly suppressed by miR-760 (Figure 3c and d). In the Ago2 immunoprecipitate, there were both circYIPF6 and miR-760 (*P* < 0.001, Figure 3e and f). Moreover, we found that biotin-labeled miR-760 WT captured significantly more circYIPF6 than biotin-labeled miR-NC and miR-760 MUT (*P* < 0.001, Figure 3g). Furthermore, we confirmed that miR-760 was decreased in glioma (*P* < 0.001, Figure 3h and i). These data demonstrated that circYIPF6 served as a sponge of miR-760 in glioma cells.

### 3.4 Inhibition of miR-760 partly reverses the regulatory effect of circYIPF6 knockdown on cell proliferation, apoptosis, and glycolysis of glioma cells

**Figure 4:**
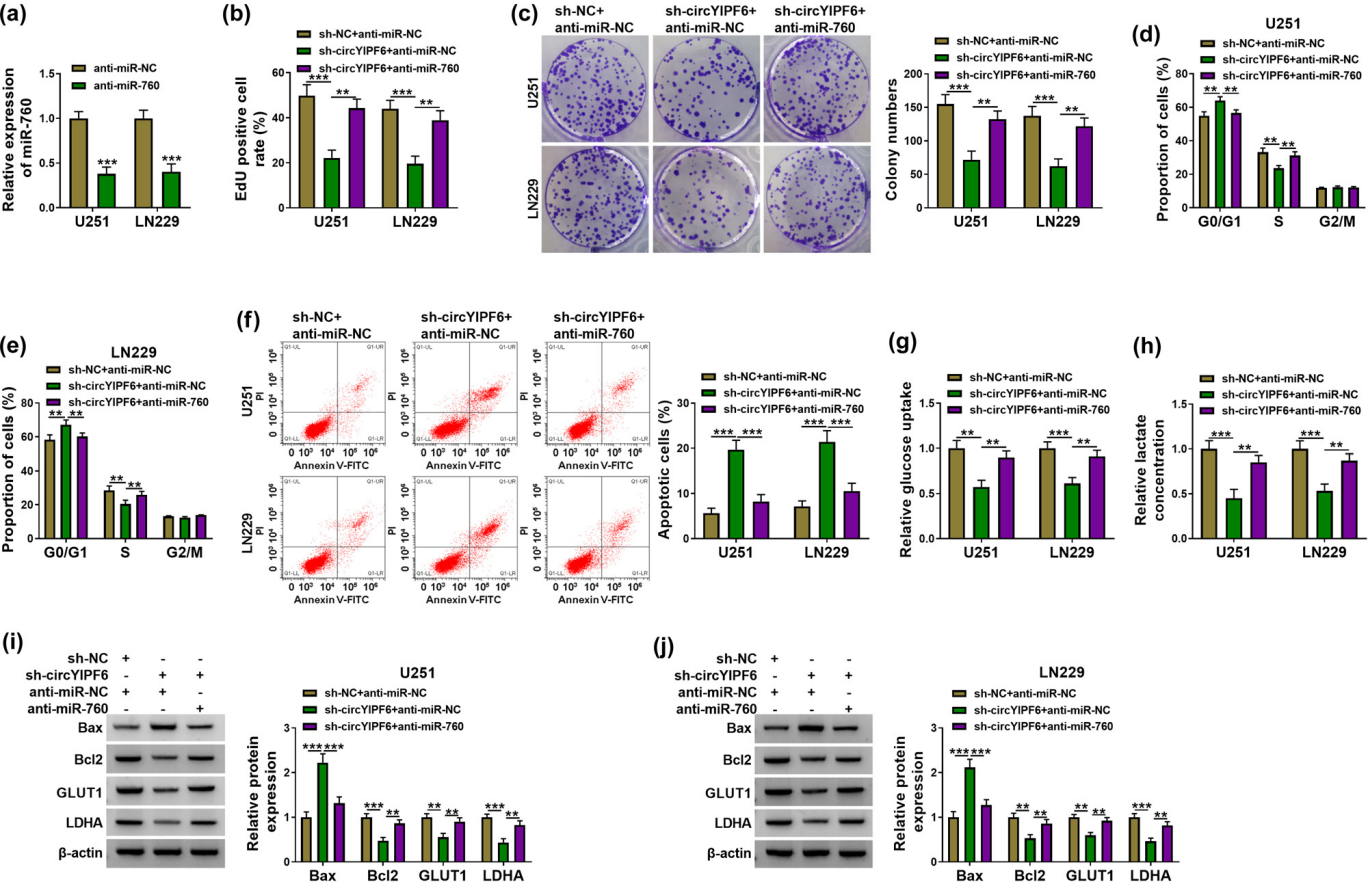
Knockdown of circYIPF6 inhibits proliferation and glycolysis and promotes apoptosis of glioma cells via sponging miR-760. (a) qRT-PCR confirmation of the miR-760 expression in miR-760 inhibitor-transfected glioma cells (*n* = 3). (b) EdU staining of glioma cells transfected with sh-NC + anti-miR-NC, sh-circYIPF6 + anti-miR-NC or sh-circYIPF6 + anti-miR-760, and EdU-positive cell rate (*n* = 3). (c) Colonies formed in glioma cells transfected with sh-NC + anti-miR-NC, sh-circYIPF6 + anti-miR-NC, or sh-circYIPF6 + anti-miR-760 (*n* = 3). (d and e) Flow cytometry detection of cell cycle distribution of glioma cells after sh-NC + anti-miR-NC, sh-circYIPF6 + anti-miR-NC, or sh-circYIPF6 + anti-miR-760 transfection (*n* = 3). (f) Flow cytometry detection of cell apoptosis of glioma cells after sh-NC + anti-miR-NC, sh-circYIPF6 + anti-miR-NC, or sh-circYIPF6 + anti-miR-760 transfection (*n* = 3). (g) Glucose uptake and (h) lactate concentration were measured in sh-NC + anti-miR-NC-, sh-circYIPF6 + anti-miR-NC-, or sh-circYIPF6 + anti-miR-760-transfected glioma cells (*n* = 3). (i and j) Western blot displaying the expression of Bax, Bcl2, GLUT1, and LDHA in glioma cells transfected with sh-NC + anti-miR-NC, sh-circYIPF6 + anti-miR-NC, or sh-circYIPF6 + anti-miR-760 (*n* = 3). ***P < 0.001, **P < 0.01.

To investigate whether miR-760 mediated the function of circYIPF6 in glioma cells, we co-transfected miR-760 inhibitors in circYIPF6-knockdown glioma cells for functional remediation experiments. miR-760 level was significantly decreased in glioma cells after transfection with miR-760 inhibitors (*P* < 0.001, Figure 4a). The results of rescue experiments exhibited that the inhibitory effects of silencing circYIPF6 on glioma cell proliferation and cell cycle were attenuated by miR-760 inhibition (*P* < 0.01, Figure 4b–e). Moreover, miR-760 inhibition significantly abolished circYIPF6 knockdown-mediated facilitation of apoptosis of glioma cells (*P* < 0.001, Figure 4f). Also, circYIPF6 knockdown repressed glucose uptake and lactate production, which were restored by anti-miR-760 (*P* < 0.01, Figure 4g and h). Western blot results showed that miR-760 inhibition also antagonized the alteration of Bax, Bcl2, GLUT1, and LDHA levels induced by circYIPF6 knockdown (*P* < 0.01, Figure 4i and j). These data revealed that the knockdown of circYIPF6 functioned tumor-suppressor effects via miR-760 in glioma cells.

### 3.5 PTBP1 is a target of miR-760 and indirectly regulated by circYIPF6

**Figure 5:**
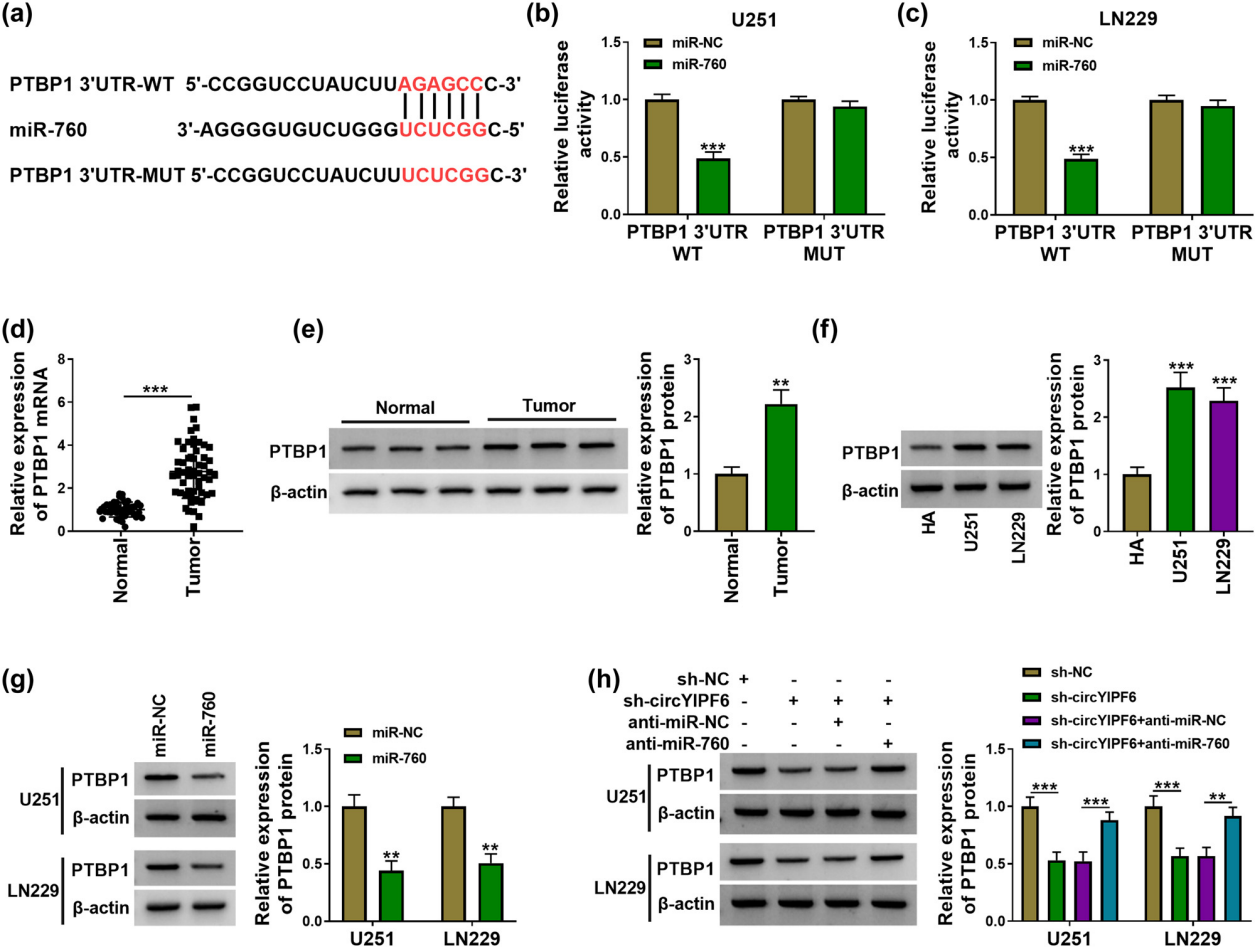
circYIPF6 regulates the expression of PTBP1 through sponging miR-760. (a) The predicted miR-760 binding sites in the 3′UTR of PTBP1 mRNA. (b and c) Luciferase reporter activity of PTBP1 3′UTR-WT or PTBP1 3′UTR-MUT in glioma cells after transfection with miR-760 (*n* = 3). (d) mRNA expression of PTBP1 in tumor tissues (*n* = 57) and normal tissues (*n* = 43) were assayed by qRT-PCR. (e) The protein level of PTBP1 in tumor tissues (*n* = 57) and normal tissues (*n* = 43) was analyzed by western blot. (f) The protein level of PTBP1 in cells was examined by western blot (*n* = 3). (g) Western blot analysis of PTBP1 protein expression after miR-760 mimics or miRNA mimics transfection (*n* = 3). (h) Western blot analysis of PTBP1 protein expression after sh-NC, sh-circYIPF6, sh-circYIPF6 + anti-miR-NC, or sh-circYIPF6 + anti-miR-760 transfection (*n* = 3). ***P < 0.001, **P < 0.01.

Subsequently, we predicted the underlying target genes of miR-760 using starBase and found that there were predicted binding sites for miR-760 in the 3′UTR of PTBP1 (Figure 5a). The dual-luciferase reporter gene assay displayed that miR-760 decreased the luciferase activity of PTBP1 3′UTR-WT (*P* < 0.001, Figure 5b and c). Furthermore, PTBP1 expression in glioma was upregulated (*P* < 0.01, Figure 5d–f). We further analyzed the regulation of miR-760 on PTBP1 level and found that overexpression of miR-760 downregulated PTBP1 expression in glioma cells (*P* < 0.01, Figure 5g). Besides, circYIPF6 silencing reduced PTBP1 protein expression, and miR-760 inhibition reversed this effect (*P* < 0.01, Figure 5h). These results suggested that miR-760 targeted PTBP1 and circYIPF6 positively regulated PTBP1 expression through acting as a miR-760 sponge.

### 3.6 circYIPF6 silencing suppresses glioma cell proliferation and glycolysis and enhances cell apoptosis through downregulating PTBP1

**Figure 6:**
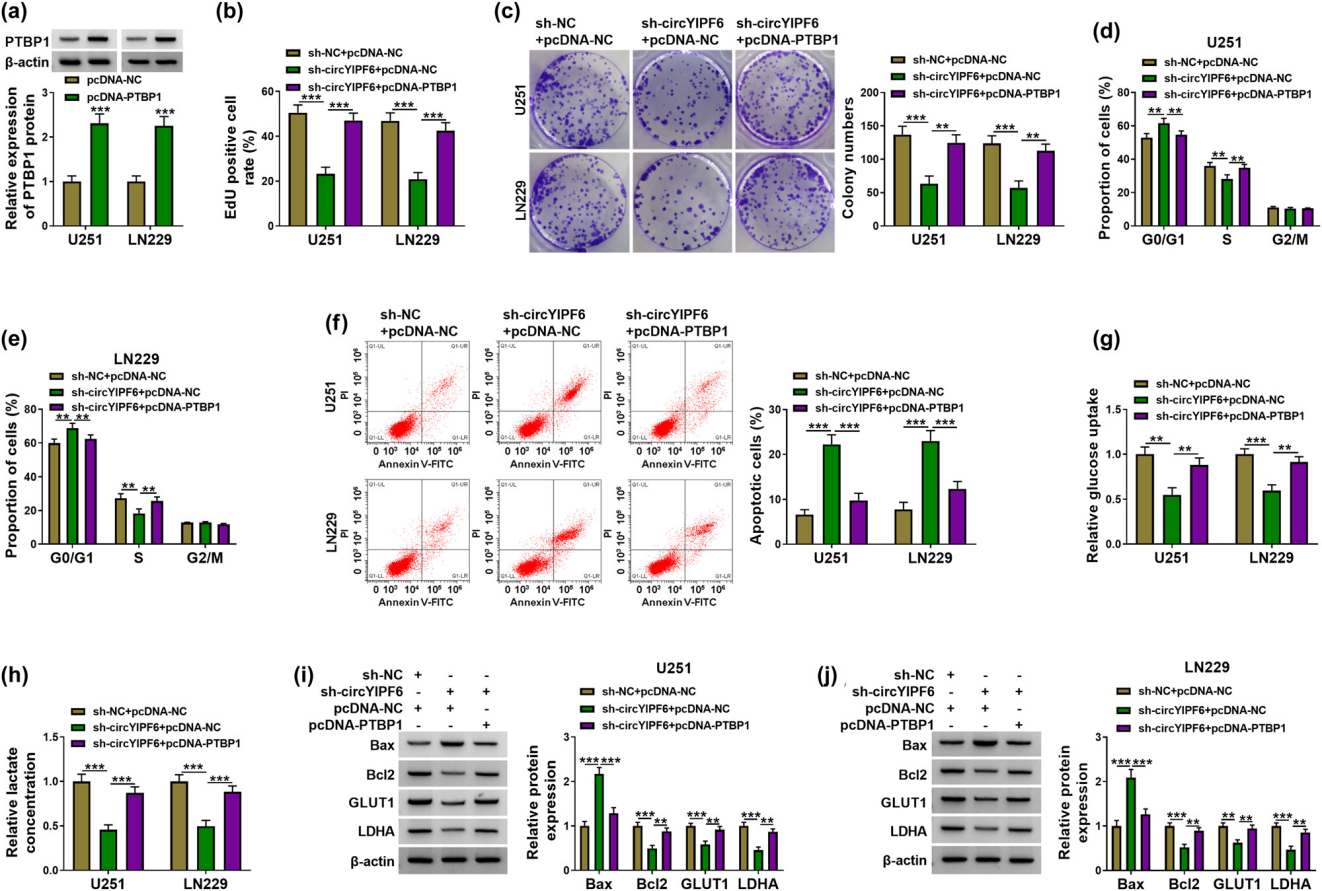
circYIPF6 silencing inhibits proliferation and glycolysis and facilitates apoptosis of glioma cells through downregulating PTBP1. (a) Western blot for the protein expression of PTBP1 in glioma cells transfected with pcDNA-NC or pcDNA-PTBP1 (*n* = 3). (b) EdU staining of glioma cells transfected with sh-NC + pcDNA-NC, sh-circYIPF6 + pcDNA-NC or sh-circYIPF6 + pcDNA-PTBP1, and EdU-positive cell rate (*n* = 3). (c) Colonies formed in glioma cells transfected with sh-NC + pcDNA-NC, sh-circYIPF6 + pcDNA-NC, or sh-circYIPF6 + pcDNA-PTBP1 (*n* = 3). (d and e) Flow cytometry detection of cell cycle distribution of glioma cells after sh-NC + pcDNA-NC, sh-circYIPF6 + pcDNA-NC, or sh-circYIPF6 + pcDNA-PTBP1 transfection (*n* = 3). (f) Flow cytometry detection of cell apoptosis of glioma cells after sh-NC + pcDNA-NC, sh-circYIPF6 + pcDNA-NC, or sh-circYIPF6 + pcDNA-PTBP1 transfection (*n* = 3). (g) Glucose uptake and (h) lactate concentration were measured in sh-NC + pcDNA-NC-, sh-circYIPF6 + pcDNA-NC-, or sh-circYIPF6 + pcDNA-PTBP1-transfected glioma cells (*n* = 3). (i and j) Western blot displaying the expression of Bax, Bcl2, GLUT1, and LDHA in glioma cells transfected with sh-NC + pcDNA-NC, sh-circYIPF6 + pcDNA-NC, or sh-circYIPF6 + pcDNA-PTBP1 (*n* = 3). ***P < 0.001, **P < 0.01.

Furthermore, we explored whether circYIPF6 performed its biological function through regulating PTBP1. As shown in Figure 6a, overexpression of PTBP1 increased the level of PTBP1 in glioma cells (*P* < 0.001). Interestingly, PTBP1 overexpression abolished the effect of circYIPF6 silencing on the proliferation (*P* < 0.01, Figure 6b–e), apoptosis (*P* < 0.001, Figure 6f), and glycolysis (*P* < 0.01, Figure 6g and h) of glioma cells. In addition, the regulation of circYIPF6 knockdown on Bax, Bcl2, GLUT1, and LDHA levels was reversed after PTBP1 over-expression (*P* < 0.01, Figure 6i and j). These data indicated that silencing of circPTBP1 blocked the deterioration of glioma cells through downregulating PTBP1, and the schematic diagram is shown in Figure 8.

### 3.7 Knockdown of circYIPF6 impedes glioma tumor growth *in vivo*

**Figure 7:**
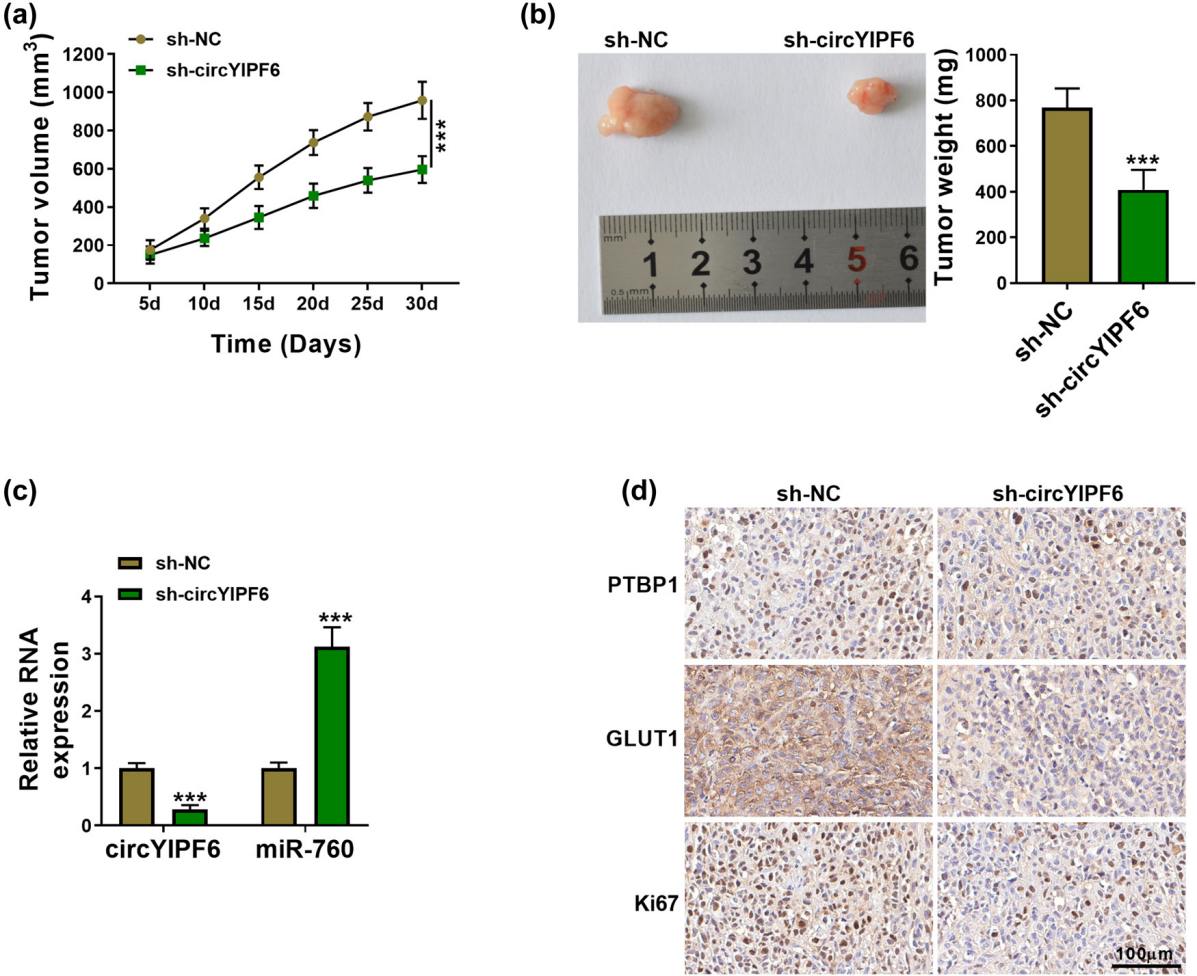
Knockdown of circYIPF6 repressed glioma tumor growth *in vivo*. (a) Tumor volume growth curve of each group (*n* = 5). (b) Representative picture (left) and tumor weight (right) of the tumors (*n* = 5). (c) The expression of circYIPF6 and miR-760 in xenograft tumors was detected by qRT-PCR (*n* = 5). (d) IHC analysis examining PTBP1, GLUT1, and Ki67 levels in xenograft tumors (*n* = 5). ***P < 0.001.

Finally, we established the xenograft tumor model by subcutaneous inoculation of U251 cells stably transfected with sh-circYIPF6 to determine the function of circYIPF6 on glioma cell growth *in vivo*. The results showed that the volume of xenografts with circYIPF6 silencing was smaller than the negative control (*P* < 0.001, Figure 7a). Consistently, the weight of xenografts was obviously reduced in circYIPF6 knocking down rather than the negative control (*P* < 0.001, Figure 7b). qRT-PCR results revealed that circYIPF6 expression was decreased and miR-760 expression was increased in xenografts with circYIPF6 silencing (*P* < 0.001, Figure 7c). In addition, the levels of PTBP1, GLUT1, and Ki67 were lower in the circYIPF6 knockdown group compared with the control group by IHC (Figure 7d). These data elucidated that silencing of circYIPF6 repressed the growth of glioma *in vivo*.

## 4 Discussion

Infinite proliferation and loss of apoptosis are major features of tumor cells. Some researchers have revealed that the differential expression of circRNA in glioma affects the cellular process [18,19,20]. Herein, we notarized that circYIPF6 was an upregulated circRNA in glioma, which is consistent with Lyu et al.’s results [10]. Functional studies uncovered that circYIPF6 silencing repressed glioma cells’ proliferation and colony formation capacity, blocked the cell cycle in G0/G1 phase, and induced cell apoptosis *in vitro*, as well as impeded glioma growth *in vivo*. However, the clinical application and value of circYIPF6 as blood biochemical indexes were not investigated in this study.

Glycolysis is the main energy source for tumor cells. Glycolysis can not only meet the large amount of energy required by the growth of malignant tumors but also the lactic acid generated during glycolysis can be transferred to the extracellular environment to acidify tumor microenvironment, which can protect tumor cells from host immune killing [21,22]. GLUT1 was a major regulatory protein of glycolysis and played a key role in mediating glucose into tumor cells [23]. Xiao et al. studies, GLUT1, an upregulated gene in prostate cancer, participated in the progression of prostate cancer via accelerating cell proliferation and glycolysis [24]. In addition, LDHA was another key glycolytic enzyme and was considered a potential anti-tumor therapeutic strategy [25]. These studies have shown that GLUT1 and LDHA could be used as markers of glycolysis in tumor cells. In our study, we monitored glucose uptake, lactate concentration, and GLUT1 and LDHA expression of glioma cells and found that the above indicators all decreased after circYIPF6 knockdown, suggesting that the knockdown of circYIPF6 suppressed glycolysis of glioma cells.

Previous researches have elucidated the function of circRNA from the view of ceRNA, such as circPTK2-repressed gastric cancer cell growth and invasion via targeting miR-196a-3p and downregulating AATK protein expression [26]. circ_0001361 accelerated cell proliferation and metastasis of lung cancer by sponge miR-525-5p to upregulate VMA21 expression [27]. circCCDC66 facilitated glioma cell migration and invasion through miR-320a/FOXM1 axis [28]. Here, we first verified that miR-760 was a target of circYIPF6. miR-760 was reported as a tumor repressor in multiple tumors [29,30,31,32]. In addition, miR-760 was reported to play the action of anti-tumor in glioma via targeting MEF2D [17]. Consistently, we detected that miR-760 was downregulated in glioma. Furthermore, our study confirmed that PTBP1 was a downstream target of miR-760 and indirectly regulated by circYIPF6. Functional rescue expression results revealed that circYIPF6 performed its role in glioma through sponging miR-760 to regulating PTBP1 expression.

**Figure 8:**
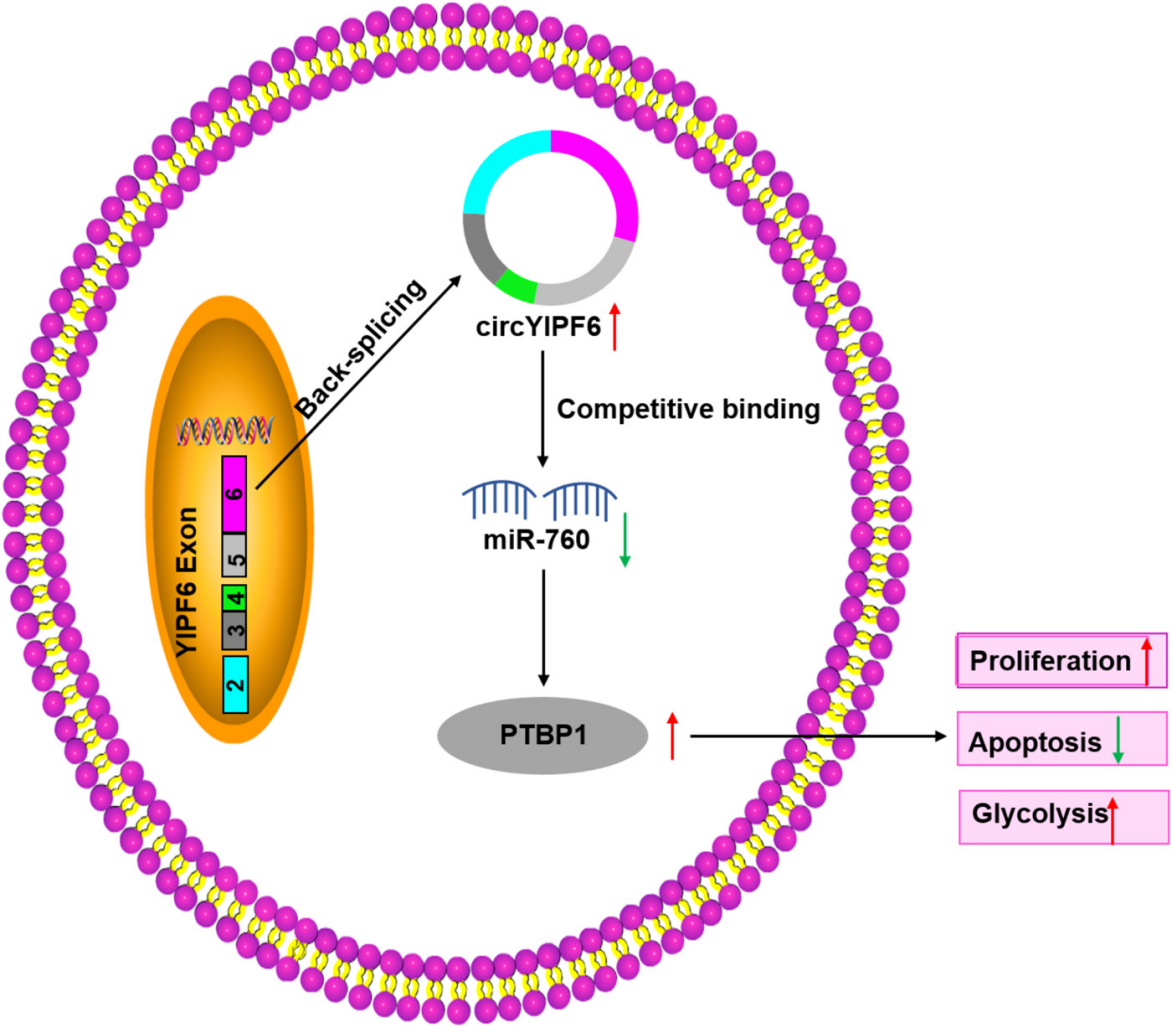
Schematic diagram illustrating the mechanism of circYIPF6 promoted glioma progression and glycolysis via miR-760/PTBP1 axis.

In summary, this study revealed a novel molecular pathway that regulated glioma progression (Figure 8), suggesting that the circYIPF6/miR-760/PTBP1 axis might be a potential therapeutic target for glioma.
